# Clustering of continuous and binary outcomes at the general practice level in individually randomised studies in primary care - a review of 10 years of primary care trials

**DOI:** 10.1186/s12874-020-00971-7

**Published:** 2020-04-15

**Authors:** Beth Stuart, Taeko Becque, Michael Moore, Paul Little

**Affiliations:** Primary Care and Population Sciences, Aldermoor Health Centre, University of Southampton, Aldermoor Close, Southampton, SO16 5ST UK

**Keywords:** Clustering, Intra-cluster correlation, Individually randomised trial, GP practice, Primary care

## Abstract

**Background:**

In randomised controlled trials, the assumption of independence of individual observations is fundamental to the design, analysis and interpretation of studies. However, in individually randomised trials in primary care, this assumption may be violated because patients are naturally clustered within primary care practices. Ignoring clustering may lead to a loss of power or, in some cases, type I error.

**Methods:**

Clustering can be quantified by intra-cluster correlation (ICC), a measure of the similarity between individuals within a cluster with respect to a particular outcome. We reviewed 17 trials undertaken by the Department of Primary Care at the University of Southampton over the last ten years. We calculated the ICC for the primary and secondary outcomes in each trial at the practice level and determined whether ignoring practice-level clustering still gave valid inferences. Where multiple studies collected the same outcome measure, the median ICC was calculated for that outcome.

**Results:**

The median intra-cluster correlation (ICC) for all outcomes was 0.016, with interquartile range 0.00–0.03.

The median ICC for symptom severity was 0.02 (interquartile range (IQR) 0.01 to 0.07) and for reconsultation with new or worsening symptoms was 0.01 (IQR 0.00, 0.07). For HADS anxiety the ICC was 0.04 (IQR 0.02, 0.05) and for HADS depression was 0.02 (IQR 0.00, 0.05). The median ICC for EQ. 5D-3 L was 0.01 (IQR 0.01, 0.04).

**Conclusions:**

There is evidence of clustering in individually randomised trials primary care. The non-zero ICC suggests that, depending on study design, clustering may not be ignorable. It is important that this is fully considered at the study design phase.

## Background

The past decade has seen a steady increase in the use of the cluster randomised trial design [1]. In cluster-randomised trials, the unit of randomisation is the cluster, such as a hospital or school, rather than the individual participant. This design is often employed when an intervention is aimed at a health practitioner level rather than an individual level or where individual randomisation is not possible [2]. Cluster randomised studies generally have lower power than individually randomised trials because there may be a correlation between the responses from participants within the same cluster. This may be due to the fact that background characteristics of participants are more similar within each cluster, and in addition, cluster-level characteristics such as the effectiveness of the practitioner may differ between clusters [2].

However, clustering may also occur in individually randomised trials, for example, the natural clustering of participants within centre in a multi-centre trial [3]. Lee and Thompson [3] reviewed individually randomised trials published in the BMJ in 2002 and found that 38/42 (90%) of them had some form of clustering. Of these, only four correctly accounted for clustering in the analysis. A failure to account for clustering in the analysis of a trial will give an unbiased estimate of the treatment effect but standard errors can be too small, leading to the type I error being too large [3, 4].

The presence of patients clustered within general practioners (GPs) or GP practices in an individually randomised trial does not necessarily imply that type I error will occur [5, 6]. Kahan and Morris [7] set out the two conditions for clustering to be ignorable:

1. The intra-cluster correlation (ICC), which represents the degree of similarity in the responses of individuals from the same cluster, must be zero; or
2. The correlation of patient assignments within clusters must be zero.

In primary care, we may expect a non-zero ICC for many outcomes. Patients are naturally clustered within GPs and practices. There may be a treatment effect of the practitioner, as the definition of “usual care” in some interventions may vary between GPs or practices. The effectiveness of some practitioners at delivering an intervention may result in clustering of patient outcomes. Individuals who choose the same GP or practice may also naturally be more similar to one another than to patients at another practice. In practice, the true value of the ICC is often not known in advance and the conservative assumption may be to assume that it will be non-zero [7].

The intervention design may also lead to the correlation of patient assignments within clusters being non-zero. This may occur in partially nested trials, for example where the intervention is delivered by a clinician, who only participates in one arm of the design [8, 9]. This correlation may also be non-zero if clusters are used in the randomisation process, for example, block randomisation, randomisation that balances on patient factors or trials where outcomes are measured at several time points [7]. Block randomisation within GP practices leads to a negative correlation between treatment assignments because for each patient assigned to one treatment arm, future patients are less likely to be assigned to that same treatment. Randomisation that stratifies on patient factors leads to correlation between treatment assignments because, for example, for each patient with high baseline severity assigned to one treatment arm, a patient with low baseline severity is less likely to be assigned to that treatment arm. These designs are common in primary care trials, particularly block randomisation and the stratification of randomisation on key patient factors.

Since both these conditions may frequently be met in primary care trials, it is possible that clustering may be non-ignorable. Moreover, even if the clustering is ignorable, but the estimates of the ICCs are high, analyses that do not adjust for clustering might lead to a loss of power [7]. It is therefore important that we have robust estimates of the ICCs that are likely to apply for different outcomes and contexts. This can help us to decide firstly whether one of the conditions for clustering being ignorable may be met and whether unadjusted analyses might lead to a potential loss of power. Estimates of the ICC may be obtained from various sources, such as previous trials, databases of ICCs or from overall patterns of ICCs. Increasingly, cluster randomised trials include estimates of the ICC, at least for the primary outcome, in their published trial reports. However, a single trial may not include the setting or outcome of interest, and precision of the ICC is often not included. Moreover, there have not been any papers which have examined ICCs for individually randomised trials.

As noted above, primary care is a logical place to examine estimates of these ICCs. This study reviewed the data from all individually randomised trials carried out in the Primary Care Research Group at the University of Southampton over the last 10 years in order to provide robust estimates of ICC values which may help to inform future studies.

## Methods

This was a secondary analysis of all individually randomised trials undertaken in the Primary Care Research Group at the University of Southampton. An audit of all trials undertaken since 2005/6 was conducted and the Principal Investigators of all identified studies were contacted to request permission to access the study data. Feasibility studies were not included as they are not designed to provide robust measures of effect size [10] and estimates of ICC from these trials may not be reliable. Ethics approval was not required for this study as analysis was based on existing datasets from previously conducted studies, all of which had the appropriate approvals in place. All data was anonymised and contained no potentially identifiable data.

For each included trial, the ICC was estimated for continuous and binary outcomes using a mixed model in Stata with GP and/or practice included as a random effect. Whilst random effects models are not appropriate for all studies, for the level of inference that was desired in all these studies it was the best approach [11].

Whether to include GP, practice or both was determined based upon the study design as set out in the study protocol and upon the data available. Using Stata version 14, it is possible to directly calculate the ICC from the stored estimates after fitting a mixed model using the command ‘*estat ICC’* [12, 13]. The ICC may be expressed as:

1 $$ \rho ={s}_b^2/\left({s}_b^2+{s}_w^2\right) $$

where ${s}_b^2$ is the between cluster component of variance and ${s}_w^2$ is the within-cluster component of variance [14]. A one-way random effects model may be written as

2 $$ {Y}_{jk}=\alpha +{e}_{jk}+{\mu}_j $$

where *Y*_*jk*_ is the observation for the *k*th individual in the *j*th cluster, *α* is a constant, *μ*_*j*_ are cluster level effects and *e*_*jk*_ are individual residual effects, and *μ*_*j*_ and *e*_*jk*_ are assumed to be normally distributed [14]. The parameters ${s}_b^2$ and ${s}_w^2$ required to calculate the ICC can be estimated from the model using restricted maximum likelihood and substituted into eq. (1).

For binary outcomes, a logistic regression model was used with a random effect for GP practice. In this case, the ICC (*ρ*_*l*_) on the logistic scale can be expressed as the proportion of the total outcome variance that is due to between-cluster variation:

3 $$ {\rho}_l={s}_b^2/\left({s}_b^2+{\pi}^{\frac{2}{3}}\right) $$

where ${s}_b^2$ is the between cluster component of variance, as in the continuous model above [14].

For each outcome, the ICC was estimated and results presented for models both with and without controlling for baseline covariates. Overall, the median ICC, interquartile range (IQR) and range for all studies and all outcomes were calculated. Where multiple studies have collected the same outcome measure, the median, IQR and range for that outcome measure were calculated.

One advantage of using mixed effects models to calculate the ICC is that covariates can be easily included. The initial analyses were performed without including any covariates. We then adjusted for sociodemographic characteristics and any potential confounders that had been included in the original analysis. This better represents the kind of analysis that would be undertaken in practice.

In order to illustrate the potential effect of clustering on the sample size, we can calculate the design effect in different situations that might arise in practice. The design effect is an adjustment made to the sample size to account for clustering in the design of a study. It is defined as the ratio of the variance of the estimator, e.g., treatment effect, when the centre effect is taken into account and the variance of the estimator assuming a simple random sample. It has been shown [8] that in a multi-centre study with two treatment arms, the design effect can be approximated by *Deff* = 1 + (*S* − 1)*ρ*, where ρ represents the ICC as defined in [1] and S is defined as

4 $$ S=\frac{n_1{n}_2}{N}{\sum}_{j=1}^Q{\left(\frac{m_{1j}}{n_1}-\frac{m_{2j}}{n_2}\right)}^2 $$

where *m*_*j*_ = *m*_1*j*_ + *m*_2*j*_ is the number of people in cluster *j*, ${n}_i={\sum}_{j=1}^Q{m}_{ij}$ is the number of people in treatment group *i*, and *N* = *n*_1_ + *n*_2_ is the total number of people in the study. The S statistic is a measure of how balanced the two randomised treatment groups are within centres. If the treatment arms are perfectly balanced (ie. an equal number of patients in both treatment arms) for every centre then S = 0, and the design effect is 1-ρ. If S < 1 (slightly unbalanced numbers of people between treatment arms in each centre), the design effect is less than 1 and the trial is overpowered. If S = 1 (somewhat unbalanced treatment arms in each centre), the design effect is equal to 1. If S > 1 (unbalanced treatment arms in each centre), then design effect> 1 and the trial is underpowered. Given that the true value of S, like the true value of the ICC, may not be known in advance, an assumed value of S > 1 may represent a conservative assumption for sample size calculations.

## Results

### Sample characteristics

A total of 17 trials had available data and were included. Although the initial plan had been to look at both GP and Practice level effects, data was only available at the GP level in two studies and therefore the analyses were limited to practice level effects.

Table 1 sets out the characteristics of included studies. There was some variability in the average cluster size, with the number of participants recruited per practice ranging from 2 to 90. This often reflected the study question; large clusters often occurred in studies aiming to recruit to large trials to answer public health questions, whilst smaller clusters tended to occur in studies of rarer conditions or with more restrictive inclusion criteria. Studies contributed between 2 and 6 outcome measures to the analyses.

**Table 1:** Study characteristics

Study ID	Study topic	Number of practices	Number of participants	Mean cluster size	Number of measures available
1	GRACE trial – antibiotics for lower respiratory tract infection [15]	67	2061	31	3
2	Internet Doctor – self management of respiratory tract infections [16]	34	3044	90	6
3	Internet based vestibular rehabilitation for dizziness [17]	55	296	5	4
4	PIPS – ibuprofen, paracetamol and steam for respiratory tract infections [18]	25	889	36	5
5	POWER plus – management of obesity [19]	56	818	15	6
6	PRIMIT – handwashing to modify respiratory tract infection transmission [20]	344	20,066	58	4
7	SNIFS – Steam inhalation & nasal irrigation for sinus symptoms [21]	72	961	13	5
8	TASTE – Probiotics and chewing gum for sore throat [22]	82	1009	11	2
9	AIRS – Autoinflation for children with otitis media [23]	39	320	8	2
10	GNOME – Topical intranasal corticosteroids in children with otitis media [24]	76	217	3	2
11	ATEAM – Alexander technique, exercise, massage for chronic back pain [25]	64	579	9	6
12	ASCOT – Antibiotics and topical nasal steroid for sinusitis [26]	58	240	4	2
13	Antibiotic prescribing for conjunctivitis [27]	30	307	10	2
14	Leaflet and antibiotic prescribing for lower respiratory tract infection [28]	37	807	22	2
15	PIPO – probiotics for prevention of otitis media in children^a^	13	267	21	2
16	THREAD – SSRI for depression [29]	115	220	2	3
17	MIBS – management of irritable bowel syndrome [30]	26	135	5	5

^a^Unpublished, private correspondence

The design of each was also evaluated to determine whether the clustering was ignorable (Table 2). To give an example, the GRACE trial of antibiotics for lower respiratory tract infection had an ICC greater than zero and had a non-zero correlation of patient assignments within GP practices due to the use of blocked randomisation. Therefore in this study, the clustering would not be ignorable. In contrast, the InternetDoctor trial of a self-management intervention for respiratory tract infections also had a non-zero ICC. However, this study used simple randomisation and therefore the correlation of patient assignment within GP practices was zero. As such clustering was ignorable for this study. In total, six studies used simple randomisation, six studies used block randomisation, three studies used randomisation stratified on patient factors and two studies used both blocked and stratified randomisation. In three studies, the ICC was zero, and in the remaining 14, the ICC was non-zero. As such in eight studies the clustering was non-ignorable, and in nine studies the clustering was ignorable.

**Table 2:** Study design – Was clustering non-ignorable?

Study ID	Study topic	Condition 1: ICC non-zero	Condition 2: Correlation of patient assignments within cluster non-zero, Reason	Both conditions met for non-ignorable clustering
1	GRACE trial – antibiotics for lower respiratory tract infection [15]	Y	Y, Block randomsiation	Y
2	Internet Doctor – self management of respiratory tract infections [16]	Y	N, Simple randomisation	N
3	Internet based vestibular rehabilitation for dizziness [17]	Y	Y, randomisation stratified on patient factors	Y
4	PIPS – ibuprofen, paracetamol and steam for respiratory tract infections [18]	Y	N, Simple randomsiation	N
5	POWER plus – management of obesity [19]	Y	Y, randomisation stratified on patient factors	Y
6	PRIMIT – handwashing to modify respiratory tract infection transmission [20]	Y	N, Simple randomsiation	N
7	SNIFS – Steam inhalation & nasal irrigation for sinus symptoms [21]	Y	N, Simple randomsiation	N
8	TASTE – Probiotics and chewing gum for sore throat [22]	Y	N, Simple randomsiation	N
9	AIRS – Autoinflation for children with otitis media [23]	Y	N, Simple randomsiation	Y
10	GNOME – Topical intranasal corticosteroids in children with otitis media [24]	N	Y, Block randomsiation	N
11	ATEAM – Alexander technique, exercise, massage for chronic back pain [25]	Y	Y, Block randomsiation	Y
12	ASCOT – Antibiotics and topical nasal steroid for sinusitis [26]	Y	Y, Block randomsiation	Y
13	Antibiotic prescribing for conjunctivitis [27]	N	Y, Block randomsiation	N
14	Leaflet and antibiotic prescribing for lower respiratory tract infection [28]	Y	Y, Block randomsiation	Y
15	PIPO – probiotics for prevention of otitis media in children^b^	Y	N, Simple randomsiation	N
16	THREAD – SSRI for depression [29]	N	Y, randomisation blocked and stratified on patient factors	N
17	MIBS – management of irritable bowel syndrome [30]	Y	Y, randomisation blocked and stratified on patient factors	Y

^b^Unpublished, private correspondence

### Clustering by GP practice

In total there were 55 outcome measures from the 17 studies for which an ICC could be calculated. Some outcomes were only collected at follow up, giving 52 outcome measures for which an ICC could be reported after controlling for baseline value of the outcome measure. The median ICC was 0.016 (IQR 0, 0.03) unadjusted and 0.011 (IQR 0, 0.026) adjusted for baseline covariates (Table 3). This suggests there is evidence clustering at the practice level. A table setting out these results split by outcome type (continuous/binary) is provided in Additional file 1.

**Table 3:** Distribution of ICCs across studies clustered by GP practice

Percentile	Unadjusted ICC (*n* = 55)	ICC adjusted for baseline characteristics (*n* = 52)
Min	0	0
1%	0	0
5%	0	0
10%	0	0
25%	0.003	0.001
50%	0.016	0.011
75%	0.030	0.026
90%	0.080	0.060
95%	0.099	0.094
99%	0.186	0.140
Max	0.186	0.140

Seven outcome measures were collected in more than one study. The median ICCs for these measures are summarised in Table 4. These suggest modest levels of clustering, largely in line with what was observed overall, with adjusted ICCs ranging from 0.00 to 0.04.

**Table 4:** ICC for outcome measures collected in multiple studies

Outcome measure	N	Unadjusted median (IQR)	Adjusted median (IQR)
Symptom severity	9	0.01 (0.00, 0.08)	0.00 (0.00, 0.03)
Reconsultation	6	0.01 (0.00, 0.08)	0.01 (0.00, 0.07)
HADS anxiety^c^	2	0.09 (0.00, 0.19)	0.04 (0.02, 0.05)
HADS depression	2	0.04 (0.02, 0.07)	0.02 (0.00, 0.05)
Patient Enablement Instrument (PEI)	2	0.02 (0.02, 0.02)	^d^
Tympanometric resolution	2	0.02 (0.00, 0.03)	0.02 (0.00, 0.03)
EQ 5D-3 L	8	0.02 (0.01, 0.03)	0.01 (0.01, 0.04)

^c^HADS Hospital Anxiety and Depression Scale

^d^PEI is only collected at follow up time points so no baseline adjustment is possible

### Effect of clustering on sample size

Depending on the balance of the treatment arms within GP practices, there may be a loss or gain of power, and the ICC will influence how large this loss or gain of power is. Table 5 presents the required sample size for various values of the ICC and S statistic likely to arise in practice. For values of S < 1, where there is slight imbalance between treatment groups within GP practices, a trial would be very slightly overpowered with an ICC of 0.01. For values of S > 1, the trial would slightly underpowered. If ICC = 0.1 (corresponding to the top 5% of the studies in our sample), then these effects would be more pronounced. However, as the true value of S may not be knowable in advance, an S value > 1 may represent a more conservative sample size assumption.

**Table 5:** Sample size required for various values of ICC and S

ICC = 0.01	Balance of treatment arms within practices	S = 0 Perfectly balanced	S = 0.75 Slightly unbalanced	S = 2 Unbalanced
	Design effect	0.99	0.9975	1.01
	Trial size			
	150	149	150	152
	300	297	299	303
	500	495	499	505
	1000	990	998	1010
	Design effect	0.9	0.975	1.1
	Trial size			
	150	135	146	165
	300	270	293	330
	500	450	489	550
	1000	900	975	1100

## Discussion

This paper presents the intra-cluster correlation coefficients from 17 individually randomised studies set in primary care. The median ICC of 0.01 was in line with previous estimates of the ICC from cluster-randomised trials, demonstrating that there is clustering of outcomes. This effect may be important in sample size calculations and the planning of analyses for individually randomised trials in primary care, depending on how the intervention will be delivered and the way in which randomisation is stratified. These are important considerations for researchers at the design phase as clustering may not be ignorable and the conservative assumption may be that the ICC is greater than 0.

### Comparison with other studies

A number of other studies have investigated intra-cluster correlation coefficients from epidemiological population based surveys [31–36]. Several authors have also looked at patterns of ICCs from studies in primary care. Adams et al. [37] undertook an analysis of cluster randomised studies and surveys in primary care. They investigated 1039 ICCs from 31 studies and found an overall ICC of 0.01, with an interquartile range 0 to 0.03. Campbell et al. [38] also investigated the factors that affect the magnitude of ICCs in various settings. Based on 145 ICCs from cluster randomised trials in primary care, they estimated a median ICC of 0.045 with range 0 to 0.28. The MRC Trial of the Assessment and Management of Older People in the Community [39], reported a median ICC was 0.016 (IQR 0.008–0.020, range 0.001–0.055) from 54 ICCs from various measures. Although these studies have concentrated on cluster randomised trials, our estimate for individually randomised trials, a median ICC of 0.01 (IQR 0.00, 0.03), is very similar.

### Strengths and limitations of the study

To our knowledge this is the first study to focus on providing estimates of the clustering in individually randomised trials in primary care. These results will be helpful to statisticians and researchers working in the design and analysis of trials in this field.

The data is limited to 17 studies run from the University of Southampton. The included studies recruited from centres across the UK and internationally, however, they were based with PIs from a single centre. This may impact on the generalisability of these results. We compared the broad areas of research represented by these trials to those reported in observational studies of GP consultations. Using International Classification of Primary Care (ICPC-2), which comprises 726 codes within 17 chapters representing bodily systems/topic areas such as musculoskeletal or circulatory conditions [40], Salisbury et al. coded 308 consultations in 22 GP practices in Bristol and North Somerset [41]. They found that the 5 most common medical reasons for consultation were Musculoskeletal (19%), Skin (8.2%), Digestive (8.2%) Respiratory (7.8%) and Psychological (7.6%). The studies included in this sample do broadly represent these conditions with Musculoskeletal (12%), Digestive (6%), Psychological (6%), Skin studies were not represented at all and Respiratory studies were over-represented (65%). It is undeniable that the included studies reflect research interests of the department, and therefore are not necessarily represented in this study in proportion to how often they are encountered in general practice. However, we feel that these estimates represent a useful starting point for those conducting research in this area and hope that other researchers will also publish estimates to allow the literature to expand to include a representative sample of ICCs.

The studies considered here were all analysed with a random effects model. Where the level of inference is the individual, a generalised estimating equation (GEE) model may be more appropriate. We have not considered the implications for these types of models and further research is needed to consider the implications, if any, for GEE models of clustering at the practice level.

We were only able to calculate the ICCs for continuous and binary outcomes, using readily available software. The calculation of ICCs for time-to-event outcomes is an area on ongoing methodological research [42].

### Implications

The implications of this study are that the amount of clustering in individually randomised trials may be similar to that of cluster-randomised trials and may not be ignorable. It is good practice to consider at the planning stages how best to take clustering into account both in the design and the analysis of trials. Where appropriate, the distribution of the ICCs presented here can be used to assist in future sample size calculations and analysis plans.

## Conclusions

In the 17 individually randomised studies in primary care, there was evidence of clustering at the practice level, with a median ICC of 0.01 (IQR 0.00 to 0.03). This is in line with ICCs previously reported in cluster-randomised trials in primary care, indicating that the amount of clustering by GP practice in individually randomised trials is at a similar level to that in cluster-randomised trials, in which GP practices are randomised rather than individual participants. This may have implications for sample size calculations. Further data from other primary care studies is required to improve generalisability.

## Supplementary information

**Additional file 1: Table S1.** Distribution of ICCs across studies clustered by GP practice for continuous and binary outcomes.

## Data Availability

The datasets analysed during the current study are not publicly available because they contain individual-level data which were obtained from third parties. Several of the participating studies may require data sharing agreements and current agreements are for the purposes of this analysis only. Please contact the corresponding author and any requests will be considered by the relevant data holder involved in this collaboration.

## Abbreviations

GEE: Generalised estimating eq.
GP: General practioner
HADS: Hospital anxiety and depression scale
ICC: Intra-cluster correlation
IQR: Interquartile range
PEI: Patient enablement instrument
