# Supplementary material for: Clustering of continuous and binary outcomes at the general practice level in individually randomised studies in primary care - a review of 10 years of primary care trials
Source: BMC Med Res Methodol. 2020 Apr 15;20:83. doi: 10.1186/s12874-020-00971-7 (PMC7158044; doi:10.1186/s12874-020-00971-7)
Supplement: Supplementary file 1 — Additional file 1: Table S1. Distribution of ICCs across studies clustered by GP practice for continuous and binary outcomes. [file 12874_2020_971_MOESM1_ESM.docx]

Additional file 1.

Table A1. Distribution of ICCs across studies clustered by GP practice for continuous and binary outcomes

|  | Continuous outcomes | | Binary outcomes | |
| --- | --- | --- | --- | --- |
| Percentile | Unadjusted ICC (n=44) | ICC adjusted for baseline characteristics (n=40) | Unadjusted ICC (n=17) | ICC adjusted for baseline characteristics (n=16) |
| Min | 0 | 0 | 0 | 0 |
| 1% | 0 | 0 | 0 | 0 |
| 5% | 0 | 0 | 0 | 0 |
| 10% | 0 | 0 | 0 | 0 |
| 25% | 0.005 | 0 | 0.003 | 0.002 |
| 50% | 0.016 | 0.008 | 0.009 | 0.016 |
| 75% | 0.036 | 0.018 | 0.016 | 0.026 |
| 90% | 0.080 | 0.052 | 0.080 | 0.070 |
| 95% | 0.099 | 0.080 | 0.088 | 0.080 |
| 99% | 0.186 | 0.140 | 0.088 | 0.094 |
| Max | 0.186 | 0.140 | 0.088 | 0.094 |

.
